# The Diagnostic Yield of Investigating Developmental Regression in Children: A Systematic Review and Meta-Analysis

**DOI:** 10.1007/s10803-025-06749-4

**Published:** 2025-02-20

**Authors:** Kirsten Furley, Audrey Teo, Katrina Williams, Mohammed Alshawsh, Amanda Brignell

**Affiliations:** 1 Department of Paediatrics, Monash University, Melbourne, VIC Australia; 2 Developmental Paediatrics, Monash Children’s Hospital, Melbourne, VIC Australia; 3 Murdoch Children’s Research Institute, Royal Children’s Hospital, Melbourne, VIC Australia; 4 Department of Paediatrics, University of Melbourne, Melbourne, VIC Australia; 5 Department of Speech Pathology, Australian Catholic University, Melbourne, VIC Australia; 6 Developmental Paediatrics, Department of Paediatrics, Monash Children’s Hospital, Level 5 Developmental Paediatrics, Clayton, Melbourne, VIC 3168 Australia

**Keywords:** Developmental regression, Children, Investigations, Diagnostic yield, Prevalence of positive finding, Genomic, Next generation genetic testing, Metabolic, Neuroimaging, Neurophysiology

## Abstract

This systematic review evaluates the diagnostic yield of investigations requested for children with developmental regression. Online databases MEDLINE, EMBASE, CINAHL, PsycINFO, Cochrane were searched to identify published records that reported a diagnostic yield for children with developmental regression. Random effects meta-analyses were performed using R software with meta package. Our search identified 11,283 published records, of which 347 were assessed for eligibility, and 15 (596 children) were included in the final systematic review and meta-analysis. Subgroup analysis assessed the diagnostic yield for investigating children with different presentations and developmental regression. Diagnostic yield results were 68% for children with neurological symptoms (two records, six children, 95%CI 32–100) and children with epileptic symptoms (two records, 56 children, 95%CI 15–100); 40% for children with neurodevelopmental delay (six records, 294 children, 95%CI 3–78); 9% for autistic children (three records, 138 children, 95%CI 0–26). Pooled analysis could not be completed for metabolic (one record, 29 children) or genetic presentations (one record, 73 children). The diagnostic yield for genetic/genomic investigations (six records, 142 children, 95%CI, 47–92) was 70%, compared with 28% for metabolic (five records, 286 children, 95%CI 0–64), 13% for neurophysiological (two records, 127 children, 95%CI 0–39) and 6% for neuroimaging (two records, 41 children, 95%CI 0–20). Investigations for children with developmental regression and neurological or epileptic symptoms resulted in the highest diagnostic yield. These results are clinically meaningful and will inform future research to advance towards an agreed investigative approach yet lack statistical significance due to small samples.

**Supplementary Information:**

The online version contains supplementary material available at 10.1007/s10803-025-06749-4.

Developmental regression refers to loss of established developmental skills. Importantly, developmental regression may be an early symptom of a serious condition that warrants early identification to guide tailored, anticipatory support for the child and family. Despite its importance as a presenting feature there is currently a lack of an agreed definition or method of measuring developmental regression. This has hindered a unified approach to recognise and characterise children from the time they begin to experience developmental regression (Barger et al., 2013; Zhang et al., 2019). A further limitation has been a focus on specific conditions that include developmental regression rather than considering regression as a symptom whilst diagnostic discovery is pursued. This means that the approach to investigation and its diagnostic yield of a child presenting with developmental regression is unclear. Lack of an agreed approach to investigate children presenting with developmental regression may result in inconsistent care and involve complex, multi-specialist assessments which adds to the diagnostic odyssey experienced by many children (Bauskis et al., 2022; Zurynski, 2017). Diagnostic clarity may assist with management decisions such as family planning and reproductive risk assessments (Zurynski et al., 2017), anticipatory care, and inform treatment options that may alter disease progression (Warmerdam et al., 2020). Diagnostic clarification is valued by parents with lived experience who report that reaching a diagnosis may enhance their ability to respond to the health, education, and disability needs of their child and to help them navigate and anticipate care needs and outcomes (Bauskis et al., 2022; Pavisich et al., 2024).

There are barriers to consistent investigation of children with developmental regression at the time a child presents (Furley et al., 2023). Their presentation is characterised by features that include age of onset of loss, the domains of skills lost, trajectories of developmental regression and co-occurring symptoms. However, at the onset of regression there may not be symptoms that would direct the clinician to request tests for a specific condition. For example, a child with regression of speech and language without seizures may not be initially investigated for DEE. Later, when seizures or verbal agnosia, often experienced by children diagnosed with Landau Kleffner syndrome, are identified, an electroencephalogram and genetic tests may be ordered.

The conditions that have historically been recognised to feature developmental regression, include (1) neurodevelopmental conditions, with autism spectrum disorder (autism) the most reported example, (2) the developmental epileptic encephalopathies, (3) inborn errors of metabolism (IEM), and (4) genetic conditions, like Rett Syndrome.

Some consider skills regression to be a common dimension experienced by many autistic children (Ozonoff & Iosif, 2019). Most commonly children who are later diagnosed as autistic lose skills in speech and social developmental domains. However, there may be no other autistic features, such as repetitive behaviours or restricted interests, at the onset of regression.

DEE diagnoses are complicated by phenotypic and genetic heterogeneity. Diagnoses are based on clinical presentations and electroencephalogram findings (EEG), with NGS is becoming an increasingly important diagnostic tool (Kaya Özçora et al., 2023).

Children with metabolic conditions causing developmental regression may initially present without systemic signs or symptoms, later signs and symptoms such as vomiting or physical exam findings of organomegaly, may become more apparent over time (Márquez-Caraveo et al., 2021).

Some conditions, such as Rett syndrome have more well described phases or patterns of skill loss that may be clinically distinguishable (Neul et al., 2010), however even within well described conditions there are emerging phenotypic expansions such as atypical Rett’s syndrome that may not be clear at the onset of symptoms. The above conditions illustrate the complexity in determining the choice of investigations. Looked at as the skill loss and other symptoms emerge, a toddler may, for example, lose the skill of being able to speak single words whilst their parents report an emergence of repetitive and routine driven behaviours. This child may be reviewed by a developmental paediatrician and diagnosed with autism and their skills loss considered to be consistent with ‘autistic regression’ with no further investigation. A primary school aged child may be less focused and have poor attention whilst experiencing loss of understanding of spoken language. They may be assessed for attention deficit hyperactivity disorder (ADHD) in general paediatrics or psychiatry and later be investigated and diagnosed with a DEE, such as LKS if their reduced ability to understand spoken language is identified. Alternatively, a toddler may regress in speech and language abilities and have deteriorating sleep and increasing agitation. Depending on who they see and whether they are referred to specialist care, they may, or may not, be referred for investigations and later be diagnosed with an IEM such as mucopolysaccharidoses type III (MPS III).

Guidelines to support clinician choice of recommendations for children presenting with symptoms of developmental regression are lacking and there are many types of investigations available for clinicians. Choice of investigation will be informed by clinician experience, subspecialty training and access to resources which may be limited, especially in low and middle income countries (Helmy et al., 2016).

Medical advancements, such as next generation sequencing (NGS), brings potential for the discovery of novel molecular findings and phenotypic expansion of conditions rarely reported to feature loss of established skills (Furley et al., 2024). NGS has also been used to identify proposed high risk candidate genes for autistic children who regress in developmental abilities (Yin et al., 2020). Access to, and use of newborn screening in the diagnostic work up for children who experience symptoms of developmental regression is not well known. Other genetic investigations, such as chromosomal microarray (CMA) have been recommended as a first line investigation for children with an intellectual disability (ID) or congenital malformations (CMA) (Carter et al., 2023), yet the diagnostic yields for children with developmental regression are rarely reported (Furley et al., 2024).

Neuroimaging is becoming increasingly sophisticated and newer techniques may offer diagnostic clarification not previously possible (Stoja et al., 2021). Indeed, there are recommendations to consider neuroimaging for children with non-syndromic developmental delays (Randhawa et al., 2022), but the role and yield of neuroimaging investigations for children with developmental regression is less clear.

There is variation is the choice of metabolic tests requested for children with neurodevelopmental disorders (Carter et al., 2023), and use and utility of testing children presenting with undifferentiated developmental regression is not known.

The diagnostic role of electroencephalogram (EEG) findings for children experiencing developmental regression is established if DEE is suspected, with well described EEG patterns of unilateral or bilateral activity over the posterior temporal region that intensify during sleep (Muzio et al., 2024) for Landau Kleffner Syndrome. However, the diagnostic role of EEG to investigate regression in autistic children (Canitano et al., 2005), or for children with undifferentiated symptoms the diagnostic role of EEG is unclear.

Identifying pathological genetic, neuroimaging, metabolic and neurophysiological findings will not only aid diagnostic clarification but also build knowledge about the underlying causal mechanisms which, in turn, could hasten therapeutic advancements. Diagnostic clarification must not overshadow the possibility of co-occurring environmental factors such as trauma (Keenan et al., 2007), or extreme nutritional deficiencies that may also cause developmental regression (Pieridou & Uday, 2023; Sharma et al., 2023).

The intention of this systematic review is to evaluate and report the diagnostic yield of investigations requested for any child with symptoms of developmental regression, thus providing important information to inform an agreed approach to investigation of developmental regression in children. This complements a comprehensive medical interview and physical examination to search for environmental and co-occurring condition risks.

## Methods

This systematic review was conducted according to the Preferred Reporting Items for Systematic Reviews and Meta- Analyses (PRISMA) guidelines (Page et al., 2021). The protocol was registered in the PROSPERO database on the 12th of July 2023 (Registration number CRD42023416466).

### Search Strategy and Data Sources

Electronic databases MEDLINE (via Ovid), EMBASE (via Ovid), CINAHL (via EBSCOhost), Cochrane and PsycINFO (via Ovid) were searched from inception to 1st of June 2023. Grey literature was searched for guidelines and academic journal and medical evidence databases (search strategy in supplementary material Table i).

### Eligibility Criteria

We included all published records of children aged up to 18 years with symptoms of developmental regression without an explanatory diagnosis. We defined developmental regression as a reported loss of an established skill/s in at least one of any of these domains; speech/language, fine and or gross motor, social, cognitive and or functional abilities. We included all study designs and excluded published records that did not report a diagnostic yield for an investigation requested for a child with developmental regression and records that only considered muscle diseases causing skills change in motor abilities.

### Study Selection Strategy and Data Extraction

**Fig. 1:**
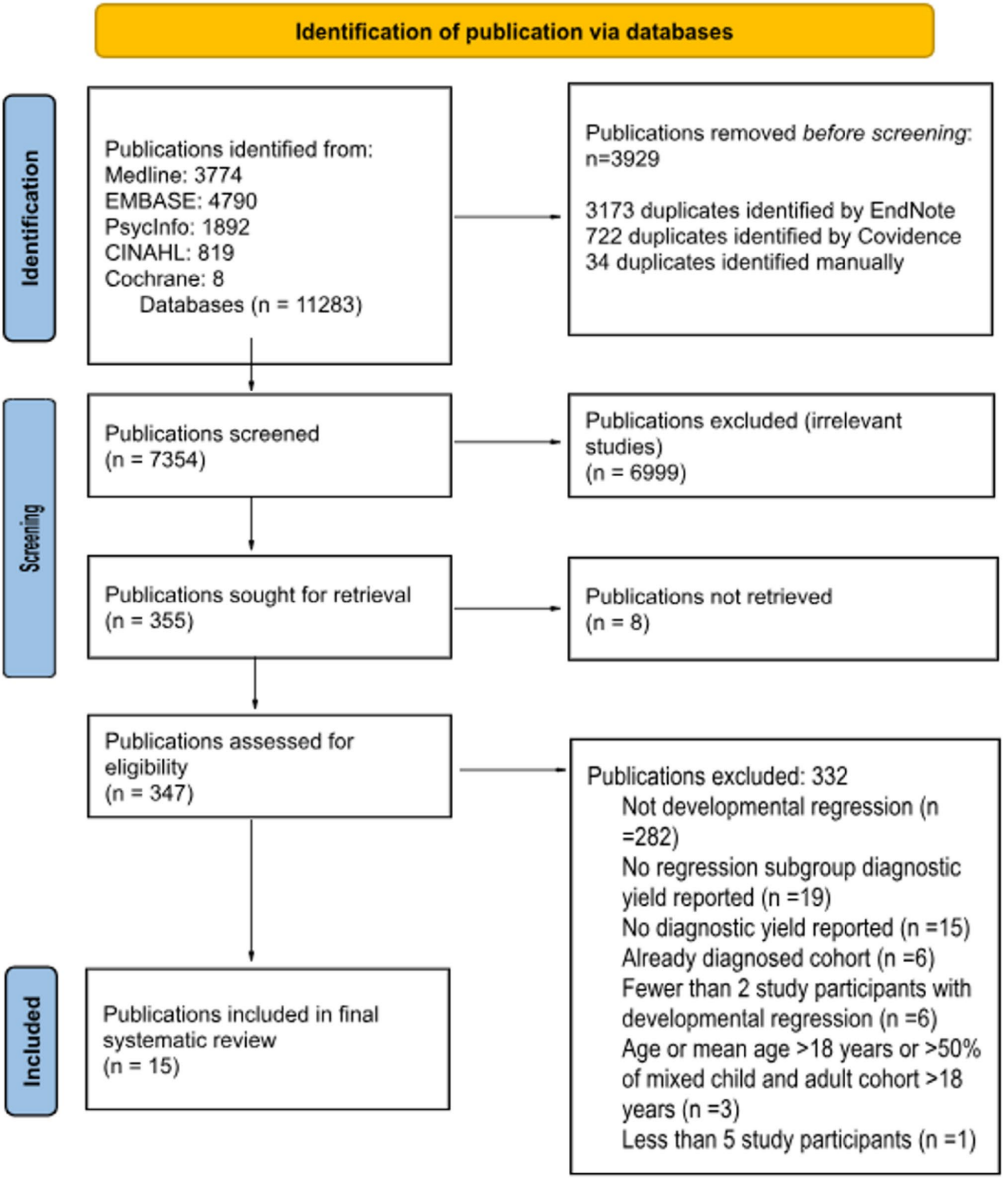
PRISMA identification of published records

Two researchers (KW, KF, AB, AT, MA) independently screened the identified titles and abstracts of included published records and those that did not meet the inclusion criteria were excluded. The full texts of remaining published records were assessed independently by two researchers (KF, AT, AB, MA) to determine eligibility for inclusion in the systematic review. Two researchers (KF, AT) extracted relevant data from each published record using a standardised form. Resolution of any disagreements in selection of published records or data extraction was achieved through discussion with a third expert reviewer. In cases where there was more than one type of investigation mentioned then data from the key or primary investigation was extracted. In cases where data was missing from selected published record, the researchers made efforts to obtain the required information by contacting the authors via email. If authors were unable to provide the missing data, the absence of the requested information in the systematic review was reported in PRIMSA Fig. 1.

### Risk of Bias Assessment

We modified the Quality In Prognosis Studies measure, QUIPS (Hayden et al., 2013) to assess risk of bias in four domains; 1.Bias due to participation; 2.Bias due to attrition; 3.Bias due to outcome measurement; and 4.Bias in statistical analysis and reporting (Table 2). At least two reviewers independently assessed risk of bias in included published records.

### Quality of Evidence

Grades of Recommendation, Assessment, Development and Evaluation (GRADE) was used to assess the certainty of evidence of outcomes (Guyatt et al., 2008). For each outcome this was rated as not serious, serious or very serious on five factors; 1.Risk of Bias; 2.Imprecision; 3. Inconsistency; 4.Indirectness; 5. Publication bias, and an overall certainty rating of low, moderate or high was calculated (Grooten et al., 2019).

### Statistical Analysis

Data was pooled using a random-effects meta-analysis using meta package (version 7.0-0) in R (version 4.3.2) and RStudio (version: 2023.12.1 Build 402), with the proportion of diagnostic yield for investigations completed for the children with developmental regression. Random-effects model considered the variation and heterogeneity in effect sizes across published records, providing a more conservative estimate of the true effect size. Statistical heterogeneity between the published records in effect measures were assessed using both the I^2^ statistic and *p* value. We considered a I^2^ value greater than 60% to represent substantial heterogeneity (Higgins et al., 2003, 2019). Clinical heterogeneity was assessed by comparing study level factors such as the number and proportion of participants investigated, types of investigation and childhood condition groups. Publication bias was evaluated using visual inspection of funnel plot and Egger’s linear regression test. Subgroup analysis was performed using meta package in RStudio and considered four subgroups; childhood condition; type of investigation; year of publication; and country/continent of study.

Childhood conditions were grouped into neurodevelopmental disability (NDD), developmental epileptic encephalopathy (DEE), suspected genetic condition-Rett syndrome, and suspected metabolic conditions- inborn error of metabolism (IEM). Grouping of conditions were based on reported study inclusions. Neurodevelopmental disorders (NDD) were further subdivided. NDD-delay referred to children who were not reaching age expected abilities in any developmental domain. Children with NDD and autism with and without developmental delays were grouped as NDD-autism; and children with NDD and undifferentiated neurological symptoms were grouped as NDD-neuro. However, some studies included children with developmental delay and intellectual disability and other symptoms/conditions such as cerebral palsy and autism in mixed neurodevelopmental cohorts (Srivastava et al., 2014). For these published studies the selected childhood condition category was based on the most prevalent symptom or condition as it was not possible to determine individual children within studies.

Children with developmental regression and unexplained neurological symptoms such as spasticity, and hypotonia were grouped as NDD-neuro based on study inclusions (Muthaffar, 2021). However, there are overlaps in symptoms across condition groups, for example a NDD-neuro study may have epilepsy as an inclusion, therefore to distinguish between DEE and NDD-neuro the studies grouped as DEE had to specify DEE in their study inclusion (Hong et al., 2020).

The type of investigations was grouped into four categories. These were 1. genetic/genomic (which included next generation sequencing (NGS), whole exome sequencing (WES) and mitochondrial disorder panels); 2. metabolic; 3. neuroimaging (magnetic resonance imaging of the brain; MRI-B); and 4. neurophysiology (electroencephalograms; EEG).

The year of publication was subdivided into four groups; 2005–2010; 2011–2015; 2016–2020; and 2021–2024. Country of published records was grouped into continents (Asia; Europe; Africa; South America and North America).

## Results

Our search strategy yielded 11,283 published records, of which 347 published records were reviewed in full text and 15 included in the final analysis. Eight full text published records were not retrievable despite attempts to contact authors (Fig. 1).

### Characteristics of the Included Published Records

Table 1 reports the characteristics of included published records. Eleven published records included children with neurodevelopmental conditions (NDD). These were sub-grouped to published records including children with developmental delay (NDD-delay, six records, total children = 294); Autistic children with and without cognitive delays, (NDD-autism, n = 3, total children = 138); and children with developmental differences and undifferentiated neurological signs or symptoms such as hypotonia or spasticity (NDD-neuro, n = 2, total children = six). The other childhood conditions were suspected developmental epileptic encephalopathy based on study use of the term DEE in their inclusion criteria, and or EEG findings and or seizures suggestive of DEE (DEE, n = 2, total children = 56); a suspected genetic condition (n = 1, total children = 73); and a suspected metabolic condition (n = 1, total children = 29). Six published records reported genetic/genomic investigations, five metabolic investigations: two neuroimaging (MRI-B) and two neurophysiological (EEG) findings. Published records were from 15 different countries grouped as Asia (n = 9), South America (n = 1), Europe (n = 3), Africa (n = 1) and North America (n = 1). Year of published records was grouped as 2005–2010 (n = 2), 2011–2015 (n = 4), 2016–2020 (n = 6), and 2021–2024 (n = 3). The age of included children ranged 3 days to 18 years and some published records reported age range, others mean or median ages.

**Table 1:**
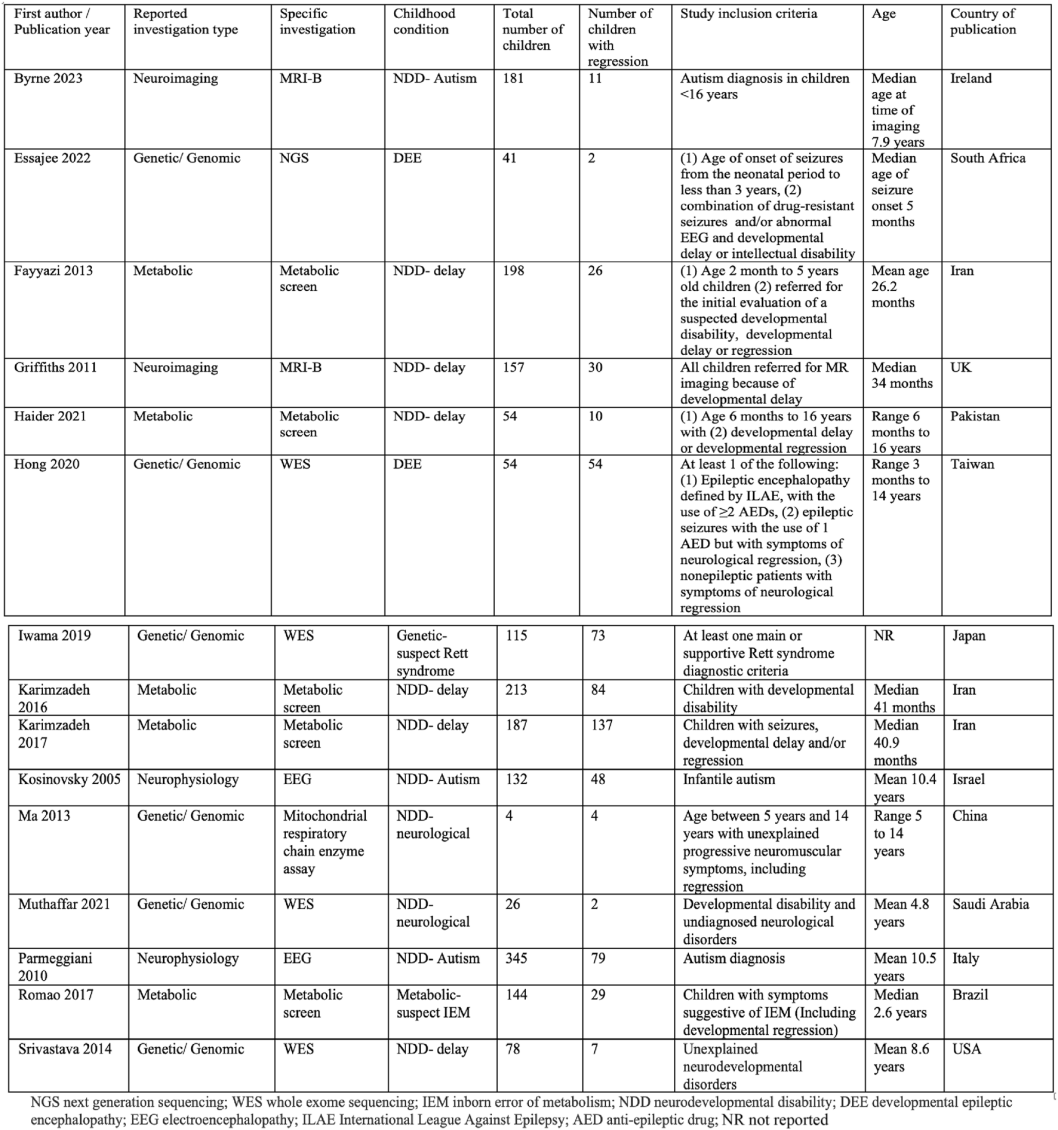
Published record characteristics

The overall risk of bias was high for published records of children with developmental regression and delay or autism or neurological symptoms, and the genetic study of suspected Rett syndrome. The two published records of children with suspected DEE were of overall low bias and the metabolic study of suspected IEM was of overall moderate risk of bias (See Table 2 Risk of Bias). Risk of Bias by type (participation, attrition, outcome measurement and statistical analysis and reporting is presented in supplementary material Figure i).

**Table 2:**
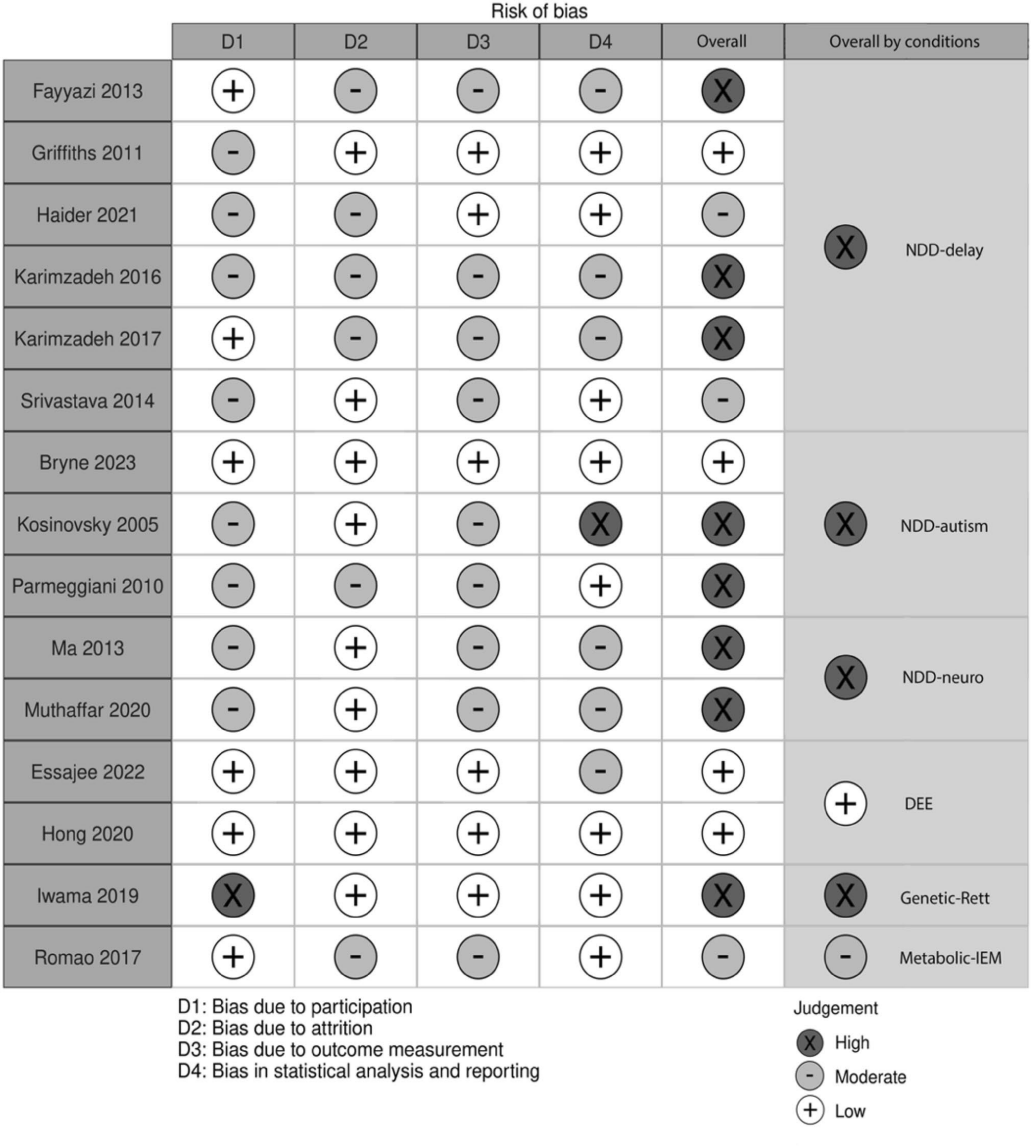
Risk of bias with overall column

There was no publication bias on visual inspection of the funnel plot and Egger’s test for publication bias yielded a *p*-value of 0.2502, exceeding the threshold of 0.05, suggesting no significant publication bias (see supplementary Figure ii and Table ii).

### Diagnostic Yield

**Fig. 2:**
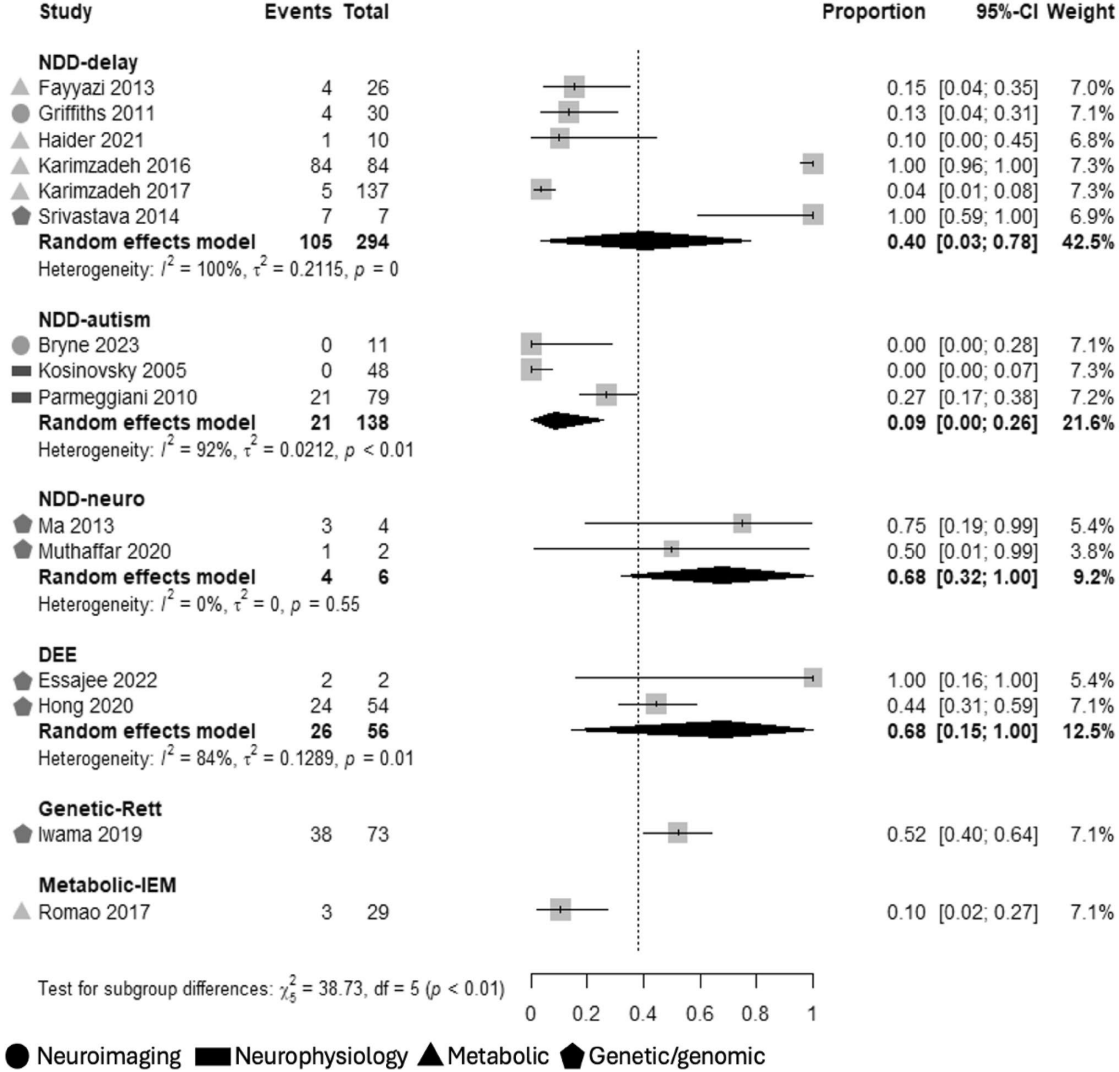
Diagnostic yield by childhood condition and investigation type

Figure 2 provides information about the diagnostic yield by childhood condition group and investigation type. As shown the diagnostic yield varied from 68% for suspected DEE conditions (95%CI 15–100) and NDD-neurological (95%CI 32–100), to 40% for NDD-delay (95%CI 3–78), and to a low of 9% for NDD-autism (95%CI 0–26). The one genetic published record of children with suspected Rett syndrome yielded a diagnosis for 52% of the 73 girls included in the study (95%CI 40–64). Investigations requested for children with suspected IEM yielded a diagnosis for 10% of the 29 included children (95%CI 2–27). Four of the six published records of children with developmental regression and NDD-delay reported on metabolic investigations, one reported on genetic/genomic investigations, and one reported on neuroimaging diagnostic yield. Two of the three published records of children with NDD-autism involved EEG investigations and one reported neuroimaging findings. The two NDD-neuro published records reported on genetic/genomic results and the DEE studies both reported on genetic/genomic results.

To investigate whether important methodological or clinical factors changed synthesised estimates, the year of publication was investigated for four time periods. Published records conducted after 2010 reported higher diagnostic yields compared to earlier records (Supplementary material Figure i). When grouped into continents, published records from Asia reported the highest diagnostic yield of 37% (95%CI 13–61), compared to Europe 14% (95%CI 0–29) (Supplementary material Figure ii).

Two records with 100% yield were included in subgroup analyses for childhood condition. One record investigated seven children with NDD-delay with genomic tests (effect size 1.0, 95%CI (59–100) (Srivastava et al., 2014). The other investigated 84 children with NDD-delay with metabolic tests (effect size 1.0, 95% CI 96–100) (Karimzadeh et al., 2016). A third published record with 100% yield was a study of 41 children with suspected DEE who underwent genomic investigations, two of whom had reported symptoms of developmental regression. Both children received an explanatory genomic result (Essajee et al., 2022). Sensitivity and meta-analysis analysis was completed for all studies (see supplementary material Table iii) and for subgroups (see supplementary material Table iv). When these published records were removed there was not a statistically significant change to the diagnostic yield results.

GRADE (Guyatt et al., 2008) was used to assess the certainty of evidence and the overall certainty reported (Iorio et al., 2015) (Table 3), with explanations of decisions reported in Table 3. Low certainty of evidence was reported for NDD-autism, and NDD-neurological due to high risk of bias and small sample sizes. Results from published records of children with NDD-delay were of moderate certainty as studies were highly heterogeneous with high rate of bias in half of the records. Results of published records of DEE were assessed to be of moderate certainty due to small sample size impacting on precision. Results of published records of genetic (suspected Rett syndrome) and metabolic (suspected IEM) published records were assessed to be of moderate certainty due to small sample sizes and high and moderate risk of bias respectively.

**Table 3:**
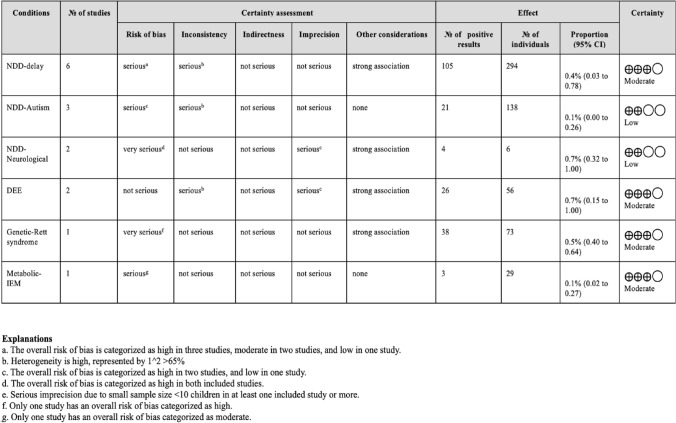
GRADE certainty of evidence

## Discussion

This is the first systematic review to report the diagnostic yield of investigating children with symptoms of developmental regression. Despite the growing awareness of conditions that feature developmental regression, and frequency of developmental regression as a symptom we only found diagnostic yield information for 596 children, from 15 published records spanning nearly 20 years (from 2005 to 2024).

When childhood conditions were grouped, children with developmental regression and neurological signs (Ma et al., 2013; Muthaffar, 2021), or with suspected DEE (Essajee et al., 2022; Hong et al., 2020), or suspected Rett syndrome (Iwama et al., 2019) were more likely to receive a diagnostic result than children investigated for an inborn error of metabolism (Romao et al., 2017), or autistic children with developmental regression (Byrne et al., 2023; Kosinovsky et al., 2005; Parmeggiani et al., 2010). There was an overall 40% diagnostic yield for children with developmental regression and developmental delay, higher than for children with suspected inborn errors or metabolism (10%) and autistic children (9%).

Developmental regression in autistic children has been studied and reported for decades yet the search for a biological marker for autistic regression remains elusive. As knowledge of phenotype characterisation advances, the heterogeneity of clinical presentation differences are highlighted. Approximately 30 percent of autistic children will regress in speech and social abilities, typically at a mean age of 20 months (Tan et al., 2021), but some autistic children will also lose skills in other domains, or at a later age. Whether later onset, or regression in domains other than speech, language and social skill presentations are phenotypic expansions of autistic regression, or novel conditions requires further scrutiny. Indeed, decisions on whether to investigate autistic children with developmental regression are not uniformly agreed, however in the latest version of the diagnostic statistical manual of mental disorders (5th ed., text rev.) authors recommend that if regression occurs in an autistic child outside the typical age range, then alternative diagnoses should be considered (American PsychiatricAssociation, 2022).

Only two published records reported on neurophysiological findings and both published records reported low diagnostic yields for autistic children with developmental regression (Kosinovsky et al., 2005; Parmeggiani et al., 2010). These results align with a recent meta-analysis on the relationship between electrophysiological (EEG) changes, epilepsy and regressive autism that reported only a weak association (Barger et al., 2017). More broadly inclusive published records are needed to determine the diagnostic utility of EEG for children with symptoms of developmental regression especially considering the significance of EEG investigations in DEE conditions (Scheffer et al., 2017). Greater phenotypic characterisation with EEG correlations may reveal specific features that increase the likelihood of significant EEG findings leading to diagnostic clarification and tailored management, for example with a diagnosis of Landau Kleffner syndrome (Clark et al., 2021).

Metabolic inborn errors of metabolism (IEM) are a heterogeneous group of disorders that can present with symptoms that include developmental regression in children. IEM’s include aminoacidopathies, organic acidaemias, lysosomal storage disorders and urea cycle disorders. Whether genomic testing such as WES, can identify all metabolic conditions that cause symptoms of developmental regression is unclear. WES is purported to be beneficial for diagnostic clarification of some inherited metabolic disorders that present with a broad range of developmental symptoms (Delanne et al., 2021), but data is not specific for children with developmental regression. We report that the pooled diagnostic yield for children with a suspected IEM was 10%, which is higher than the diagnostic yield of metabolic testing reported for children with ID or GDD (Vallance et al., 2021). Early detection of metabolic conditions is essential to ensure access to tailored interventions that may be disease modifying and improve neurodevelopmental outcomes (Eun & Hahn, 2015).

We report that genetic/genomic investigations were most likely to result in an explanatory diagnosis regardless of childhood condition (Table V in supplementary material). Genomic investigations, such as WES, are considered especially important for diagnosing clinically heterogeneous presentations such as are commonly reported for children with developmental regression (Iglesias et al., 2014). WES was reported to be first used as a clinical tool in 2009 and improvements to access and affordability have contributed to the global increase in availability (Phillips et al., 2021), and clinical use over the last decade. However, health inequity exists in accessing genomic testing especially in lower- and middle-income countries (Helmy et al., 2016). In 2020 in Australia the national health insurance scheme, Medicare, introduced a rebate to cover the cost of WES for children under the age of 10 years who met specified criteria including moderate to severe intellectual disability (ID), or global developmental delay (GDD) and or facial dysmorphism and or congenital abnormalities (Sachdev et al., 2021). Currently, children without an ID or GDD or children over the age of 10 years who regress in developmental abilities are not able to access Medicare funding for whole exome sequencing. This review describes the characteristics of included studies which report that the age of children presenting with developmental regression may be older than 10 years and children may not have an ID or GDD. The utility of genomic testing for children with undifferentiated developmental regression is high given diagnostic clarification may result in important changes to management (Tan et al., 2017). For example, in one included study a child with developmental regression and symptoms suggestive of Leigh syndrome had WES testing and a diagnosis of sulfite oxidase deficiency was made, with a direct influence on a management change (Hong et al., 2020). There are health economic benefits to investigating complex presentations with WES over a series of potentially invasive tests and procedures (Schofield et al., 2019). Indeed, WES has been recommended as first line investigation for children with unexplained neurodevelopmental disorders (Srivastava et al., 2019).

The role of neuroimaging for children with developmental regression remains unclear. Both published records that reported MRI-Brain results included in this meta-analysis reported a low diagnostic yield. Similarly, results from a recent systematic review that aimed to report rates of MRI abnormalities in children with developmental delays found that there were widely varying rates of abnormality depending on clinical presentations and insufficient evidence to inform guideline recommendations (Murias et al., 2017). Further published records that consider the symptoms of developmental regression and include potentially differentiating features such as age of onset of regression, types of skills lost and trajectory of loss, and any co-occurring symptoms or clinical findings are essential to determine the diagnostic yield of MRI-Brain specifically for children with developmental regression.

Diagnostic yield varied by year of publication and country/continent of publication (see supplementary material Figure iv). In many countries over the last decade investigations such as genomic testing has become more accessible. We report that published records before 2011 had a diagnostic yield of 13% and neither record included genetic/genomic investigations (Kosinovsky et al., 2005; Parmeggiani et al., 2010). When published records were grouped into continents, the most records were from Asia and were mostly genetic/genomic or metabolic investigations. The use of investigations, such as genomic investigations, and availability of newborn screening tests may vary between countries, and influence choice of investigations. Infants identified through newborn screening may be diagnosed with a condition known to feature developmental regression, changing timing of interventions and improving outcomes. One example is X-linked adrenoleukodystrophy, which is included in newborn screening in many states of the United States of America (Reynolds et al., 2023) that typically presents with developmental regression in later primary school age. Earlier detection and intervention may alter disease progression and enable child and family support network establishment (Moser et al., 2005). Currently in Australia the inclusion of X-linked adrenoleukodystrophy to the panel of conditions screened in newborns is under review with the medical services advisory committee.

## Limitations

Grouping childhood conditions aimed to determine whether developmental abilities, co-occurring conditions like autism or seizures, influence the diagnostic yield for children presenting with developmental regression. However, as described, there are overlaps between condition groups that may have impacted upon reported diagnostic yields. For example, a recent study reported outcomes for 78 children with neurodevelopmental disorders, of whom 7 experienced developmental regression. Of these, all 7 received a diagnostic outcome from whole exome sequencing; 2 children were diagnosed with Phelan McDermid syndrome, 1 with Rett syndrome, 3 with neurogenetic conditions and 1 with DEE. For the child diagnosed with DEE the study did not report clinical details of symptoms prior to or at the time of regression onset. The children with neurogenetic conditions had undifferentiated neurological signs such as diplegia which overlaps with NDD-neuro grouping however whether these signs were present at the onset of regression is unclear (Srivastava et al., 2014). Another study included 187 children with developmental delay with or without regression and seizures. The authors did not report overlapping symptoms, therefore it was not possible to determine how many of the 137 children with regression also presented with seizures (Karimzadeh et al., 2017). Future research needs to report clinical characteristics at the onset of developmental regression to enable clinicians to request investigations based on evidence informed diagnostic yield data tailored to clinical characteristics.

Lastly, there is no agreed approach to define developmental regression and therefore future research must reach towards a consensus to enable consistency across studies that include children experiencing developmental regression.

### Clinical Impacts

Children with symptoms of developmental regression will benefit from being identified and linked in with supportive services early, regardless of their eventual diagnosis. Investigations are essential to find a cause, inform supportive and anticipatory care prognosticate and, in some cases, allow for early access to therapeutics with potential to alter disease progression. Consistency in investigative approach will increase diagnostic clarity with far-reaching impacts for child and family outcomes.

Children who experience developmental regression with neurological signs such as hypotonia, or suspected DEE based on the presence of seizures and or suggestive EEG findings, were more likely to receive a diagnosis when compared to children with symptoms of developmental regression with developmental delays, autism or suspected IEM.

Regardless of childhood condition this systematic review supports early consideration of genetic and genomic investigations for any child presenting with symptoms of developmental regression when compared to metabolic, neurophysiology or neuroimaging investigations. We acknowledge that health inequity is a barrier for some countries that do not have access to newborn screening or genetic/genomic technologies, and future research should consider availability and acceptability of investigations.

More published records are needed to refine investigations for other clinical characteristics that co-occur with developmental regression, like the age of onset of regression, types of skills lost, pre loss developmental abilities, dysmorphic or congenital features and onset of intellectual disability. However, evidence from developmental disability presentations more broadly suggest factors such as intellectual disability and congenital abnormalities will also increase diagnostic yield. Despite the frequency of developmental regression as a symptom, there is currently limited good quality data and insufficient evidence to inform guideline recommendations.

## Conclusions

Publications that report the diagnostic yield of investigating children with symptoms of developmental regression are scarce. Despite the small number of published records and sample size, children with developmental regression and undifferentiated neurological or epileptic symptoms are most likely to receive an informative diagnosis. Diagnostic yield of genetic and genomic investigations were statistically significantly higher when compared with metabolic, neuroimaging and neurophysiology investigations for any child presenting with symptoms of developmental regression.

These results have identified knowledge gaps that need to be addressed to allow immediate translation of study findings to improve care for children with developmental regression. Namely, studies including children with developmental regression should report clinical characteristics at presentation so that the diagnostic yield of investigations completed early are known and this must occur in parallel with an agreed approach to define and measure developmental regression in children.

## Supplementary Information

Below is the link to the electronic supplementary material.

Supplementary file1 (DOCX 290 KB)

## Abbreviations

DEE: Developmental Epileptic Encephalopathy
IEM: Inborn Error of Metabolism
NGS: Next generation sequencing
WES: Whole exome sequencing
ES: Exome sequencing
EEG: Electroencephalogram
MRI-B: Magnetic resonance imaging of the brain
NDD: Neurodevelopmental disability
