# Supplementary material for: The Diagnostic Yield of Investigating Developmental Regression in Children: A Systematic Review and Meta-Analysis
Source: J Autism Dev Disord. 2025 Feb 20;56(7):2721–34. doi: 10.1007/s10803-025-06749-4 (PMC13346241; doi:10.1007/s10803-025-06749-4)

Supplementary Table i) Search strategy Ovid MEDLINE(R) 1946 to May 31, 2023

| # | Query |
| --- | --- |
| 1 | developmental disabilities/ |
| 2 | neurodevelopmental disorders/ |
| 3 | Neurocognitive Disorders/ |
| 4 | Mental Retardation, X-Linked/ or Intellectual Disability/ |
| 5 | Neurodegenerative Diseases/ |
| 6 | Epileptic Syndromes/ |
| 7 | Child Development Disorders, Pervasive/ |
| 8 | exp Autism spectrum disorder/ or Autistic Disorder/ |
| 9 | (autis* or asperger* or intellectual disabilit* or genetic disorder* or metabolic disorder*).ti,ab. |
| 10 | 1 or 2 or 3 or 4 or 5 or 6 or 7 or 8 or 9 |
| 11 | (regress* or deteriorat* or decline or loss or setback or retard*).ti,ab. |
| 12 | (regress* adj1 (poisson or linear or univar* or multi* or adjust* or unadjust* or logis* or analysis)).ti,ab. |
| 13 | (regress* adj1 (caudal or spinal)).ti,ab. |
| 14 | 10 and 11 |
| 15 | 12 or 13 |
| 16 | 14 not 15 |
| 17 | ((neurodev* or neuro-dev* or neurodegen* or development* or neurolog* or mental or autis*) adj1 (regress* or deteriorat* or decline or loss or setback or retard*)).ti,ab. |
| 18 | ((skill* or milestone* or ability* or function) adj1 (regress* or deteriorat* or decline or loss)).ti,ab. |
| 19 | (developmental regression or neurodevelopmental regression or regression in development or developmental stagnation or developmental delay* or neurodevelopmental delay*).ti,ab. |
| 20 | ((progressive intellectual and neurological deterioration) or PIND or childhood dementia or childhood disintegrative disorder).ti,ab. |
| 21 | 16 or 17 or 18 or 19 or 20 |
| 22 | Magnetic Resonance Imaging/ |
| 23 | Magnetic Resonance Spectroscopy/ |
| 24 | Cytogenetic Analysis/ |
| 25 | Karyotyping/ |
| 26 | In Situ Hybridization, Fluorescence/ |
| 27 | Molecular Diagnostic Techniques/ |
| 28 | Diagnos*.ti,ab. |
| 29 | (neuroimaging or imaging or magnetic resonance or MRI or tomography or CT scan).ti,ab. |
| 30 | (chromosome* or variant* or alleles* or genetic* or genomic or gene* or panel or exome* or sequencing or NGS or X-linked or Fragile X or phenotype* or cytogenetic* or Karyotyp* or FISH Techni* or Fluorescence in Situ Hybridization).ti,ab. |
| 31 | 22 or 23 or 24 or 25 or 26 or 27 or 28 or 29 or 30 |
| 32 | ((diagnos* or investigat* or test* or screen*) adj2 (yield* or finding* or result* or outcome* or prevalence or accuracy or usefulness or performance)).ti,ab. |
| 33 | ((screening or investigat* or test*) adj5 (finding* or result* or outcome* or disorder* or diagnos*)).ti,ab. |
| 34 | Predictive Value of Tests/ |
| 35 | "Sensitivity and Specificity"/ |
| 36 | (yield* or diagnostic yield or prevalence of investigation or prevalence or proportion or incidence of yield or prevalence of positive result* or risk of positive finding* or diagnostic performance or diagnostic accuracy or positive predictive value or negative predictive value or predictive value or false-positive* or false-negative*).ti,ab. |
| 37 | 32 or 33 or 34 or 35 or 36 |
| 38 | (newborn* or new-born* or baby or babies or neonat* or neo-nat* or infan* or toddler* or pre-schooler* or preschooler* or kinder or kinders or kindergarten* or kinder-aged or boy or boys or girl or girls or child or children or childhood or pediatric* or paediatric* or school-age* or schoolage* or schoolchild* or schoolgirl* or schoolboy* or adolescen* or youth or youths or teen or teens or teenage*).af. |
| 39 | 21 and 31 and 37 and 38 |
| 40 | limit 39 to humans |
| 41 | limit 40 to English language |

Supplementary Table ii) Egger’s linear regression test

| >metabias (x, method.bias = “linreg”)  Linear regression test of funnel plot asymmetry  Test result: t = -1.20, df = 13, p-value = 0.2502  Bias estimate: -8.1458 (SE = 6.7672) | Details  -multiplicative residual heterogeneity variance (tau2 = 394.2761)  -predictor: standard error  -weight: inverse variance |
| --- | --- |

*P*-value <0.05: Possible publication bias

*P*-value >0.05: No publication bias

Supplementary Table iii) Sensitivity analysis of the meta-analysis results of the overall investigation yield

| **No** | **Study excluded** | **Proportion** | **95%-CI** | **I^2^ (%)** |
| --- | --- | --- | --- | --- |
| 1 | Omitting Bryne 2023 | 0.4097 | [0.2094; 0.6099] | 99.8% |
| 2 | Omitting Essajee 2022 | 0.3442 | [0.1536; 0.5349] | 99.8% |
| 3 | Omitting Fayyazi 2013 | 0.3986 | [0.1928; 0.6043] | 99.8% |
| 4 | Omitting Griffiths 2011 | 0.4002 | [0.1949; 0.6055] | 99.8% |
| 5 | Omitting Haider 2021 | 0.4018 | [0.1977; 0.6058] | 99.8% |
| 6 | Omitting Hong 2020 | 0.3770 | [0.1688; 0.5852] | 99.8% |
| 7 | Omitting Iwama 2019 | 0.3709 | [0.1637; 0.5782] | 99.8% |
| 8 | Omitting Karimzadeh 2016 | 0.3265 | [0.1474; 0.5056] | 95.5% |
| 9 | Omitting Karimzadeh 2017 | 0.4077 | [0.2059; 0.6096] | 99.7% |
| 10 | Omitting Kosinovsky 2005 | 0.4104 | [0.2102; 0.6105] | 99.6% |
| 11 | Omitting Ma 2013 | 0.3595 | [0.1598; 0.5593] | 99.8% |
| 12 | Omitting Muthaffar 2020 | 0.3764 | [0.1752; 0.5776] | 99.8% |
| 13 | Omitting Parmeggiani 2010 | 0.3906 | [0.1824; 0.5988] | 99.8% |
| 14 | Omitting Romao 2017 | 0.4024 | [0.1980; 0.6068] | 99.8% |
| 15 | Omitting Srivastava 2014 | 0.3309 | [0.1483; 0.5134] | 99.8% |
| Pooled estimate | | 0.3805 | [0.1871; 0.5740] | 99.8% |

Supplementary Table iv) Sensitivity analysis of the meta-analysis results of the subgroups analysis

| **Conditions** | **Study excluded** | **Proportion** | **95%-CI** | **I^2^ (%)** |
| --- | --- | --- | --- | --- |
| NDD-delay | Omitting Fayyazi 2013 | 0.4544 | [0.0158; 0.8930] | 99.9% |
|  | Omitting Griffiths 2011 | 0.4588 | [0.0228; 0.8947] | 99.9% |
|  | Omitting Haider 2021 | 0.4639 | [0.0336; 0.8942] | 99.9% |
|  | Omitting Karimzadeh 2016 | 0.2818 | [0.0000; 0.6312] | 96.8% |
|  | Omitting Karimzadeh 2017 | 0.4796 | [0.0605; 0.8988] | 99.0% |
|  | Omitting Srivastava 2014 | 0.2882 | [0.0000; 0.6468] | 99.9% |
|  | Pooled estimate | 0.4045 | [0.0329; 0.7761] | 99.8% |
| NDD-autism | Omitting Bryne 2023 | 0.1287 | [0.0000; 0.3890] | 96.2% |
|  | Omitting Kosinovsky 2005 | 0.1345 | [0.0000; 0.3950] | 91.8% |
|  | Omitting Parmeggiani 2010 | 0.0000 | [0.0000; 0.0273] | 0.0% |
|  | Pooled estimate | 0.0866 | [0.0000; 0.2585] | 92.5% |

Supplementary Figure i) Risk of bias


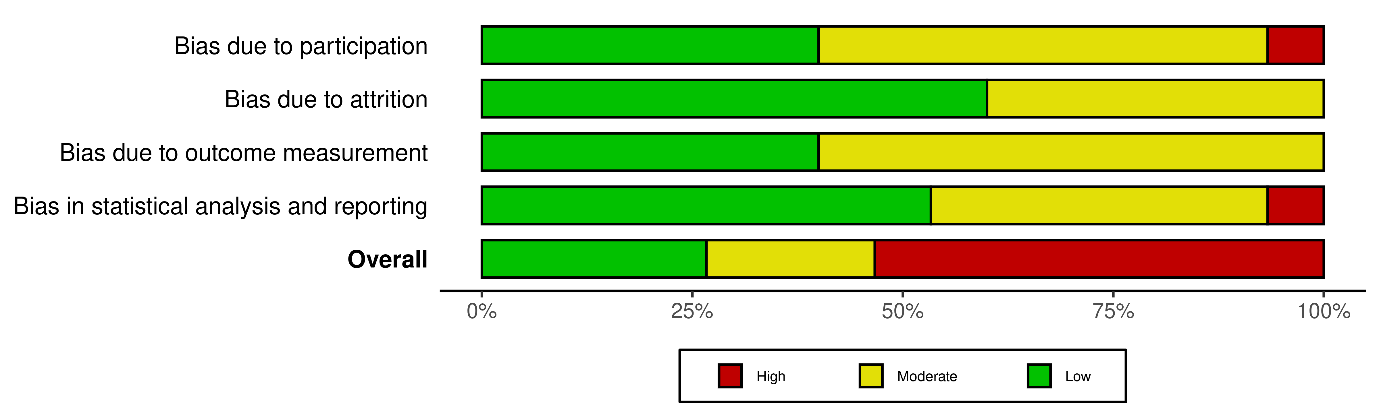


Supplementary Figure ii) Funnel plot publication bias
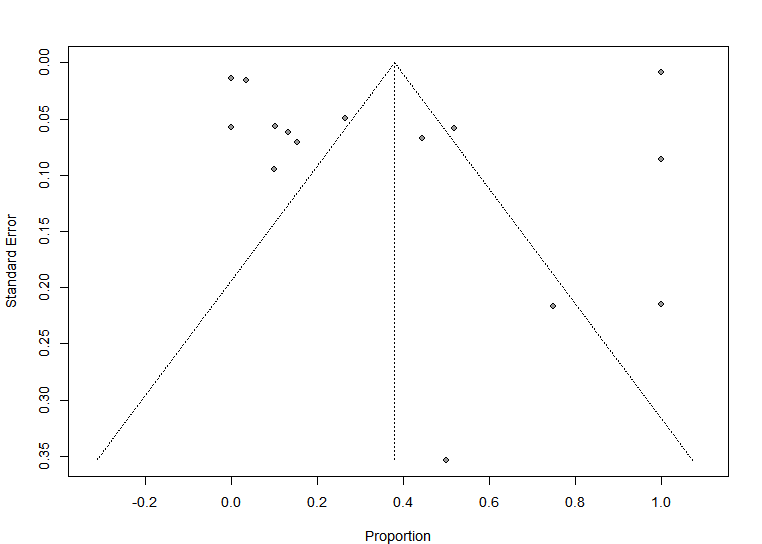


Supplementary Figure iii) Diagnostic yield by year of publication


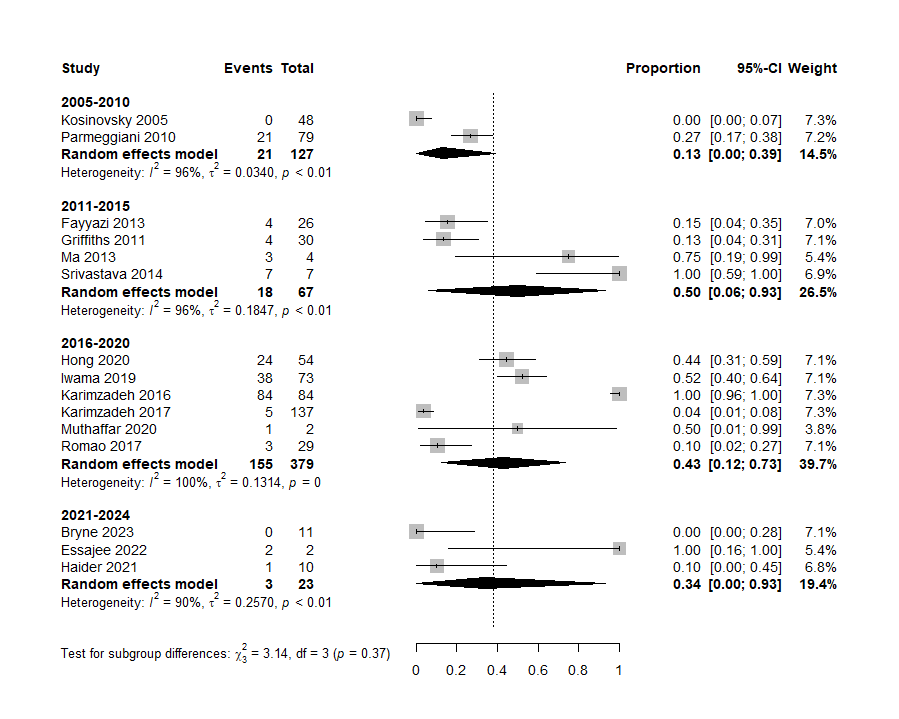


Supplementary Figure iv) Continent of study


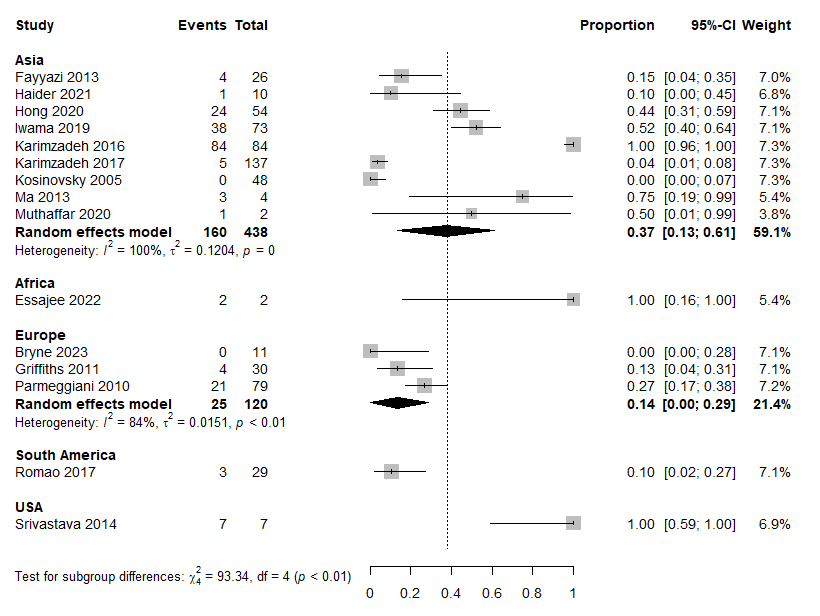


Supplementary figure V) Diagnostic yield by type of investigation


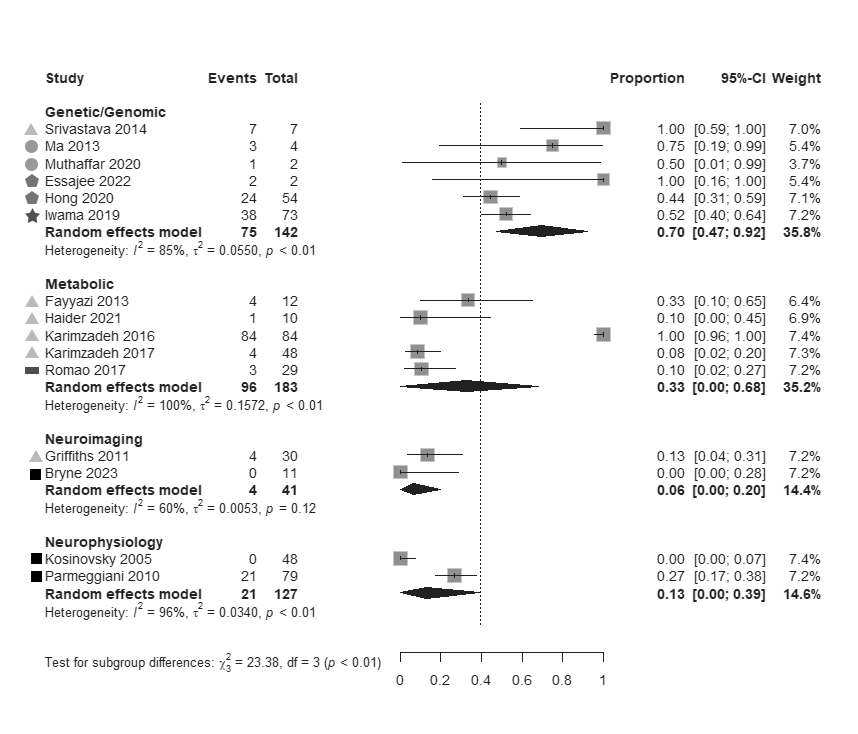


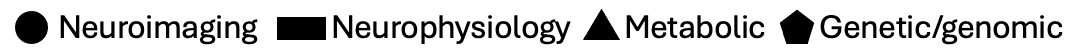

Supplement: Supplementary file 1 — Supplementary file1 (DOCX 290 KB) [file 10803_2025_6749_MOESM1_ESM.docx]
